# Supplementary material for: Optimal age-specific vaccination control for COVID-19: An Irish case study
Source: PLoS One. 2023 Sep 5;18(9):e0290974. doi: 10.1371/journal.pone.0290974 (PMC10479919; doi:10.1371/journal.pone.0290974)
Supplement: S1 Table — (PDF) [file pone.0290974.s001.pdf]

# Table of notations

| **Model states** | **Symbol** |
|---|---|
| Susceptible not yet vaccinated (o65 and y65) | $S_O, S_Y$ |
| Received vaccine, waiting for it to take effect (o65 and y65) | $V_O, V_Y$ |
| Received vaccine but was not effective (o65 and y65) | $N_O, N_Y$ |
| Susceptible, refusing or unable to receive vaccine (o65 and y65) | $U_O, U_Y$ |
| Exposed (o65 and y65) | $E_O, E_Y$ |
| Infectious (o65 and y65) | $I_O, I_Y$ |
| Recovered (o65 and y65) | $R_O, R_Y$ |
| Protected from vaccine (o65 and y65) | $P_O, P_Y$ |
| Total number of people in age group (o65 and y65) | $T_O, T_Y$ |
| **Model Parameters** | **Symbol** |
| Rate at which an o65 person infects an o65 person | $\beta_{OO}$ |
| Average number of o65 people infected by an o65 person | $R0_{OO}$ |
| Rate at which a y65 person infects an o65 person | $\beta_{YO}$ |
| Average number of o65 people infected by a y65 person | $R0_{YO}$ |
| Rate at which an o65 person infects a y65 person | $\beta_{OY}$ |
| Average number of y65 people infected by an o65 person | $R0_{OY}$ |
| Rate at which a y65 person infects a y65 person | $\beta_{YY}$ |
| Average number of y65 people infected by a y65 person | $R0_{YY}$ |
| Rate at which exposed becomes infected | $\gamma_E$ |
| Rate at which infected becomes recovered | $\gamma_I$ |
| Rate at which vaccinated becomes protected | $\gamma_V$ |
| Vaccine effectiveness | $\alpha_V$ |
| Percentage of over 65s refusing the vaccine | $r_O$ |
| Percentage of under 65s refusing the vaccine | $r_Y$ |
| **Control functions** | **Symbol** |
| Percentage of over 65s to get vaccinated at time $t$ | $u_O(t)$ |
| Percentage of under 65s to get vaccinated at time $t$ | $u_Y(t)$ |
| **Optimisation Functions and Parameters** | **Symbol** |
| Hamiltonian function | H |
| Cost function | $\mathcal{F}$ |
| Age specific weight constants | $W_O, W_Y$ |
