# Supplementary material for: Optimal age-specific vaccination control for COVID-19: An Irish case study
Source: PLoS One. 2023 Sep 5;18(9):e0290974. doi: 10.1371/journal.pone.0290974 (PMC10479919; doi:10.1371/journal.pone.0290974)
Supplement: S1 Appendix — (PDF) [file pone.0290974.s002.pdf]

Appendix A

Existence of an optimal solution

The system of ODEs describing the system dynamics can be written in compact form as

$$g(t,x,u) := \frac{dx(t)}{dt} \tag{0.1}$$

where x denotes the variable vector
$x = (\ S_O, V_O, N_O, U_O, E_O, I_O, R_O, P_O, S_Y, V_Y, N_Y, U_Y,\ E_Y, I_Y, R_Y, P_Y\ )$. Firstly, we assume g satisfies the following:

(a) $|g(t,x,u)| \leq C_1(1+|x|+|u|)$

(b) $|g(t,x,u) - g(t,x',u)| \leq C_2|x-x'|(1+|u|)$

for some positive constants C_1, C_2 and for all t, x, x' and $u \in \Omega$. As stated in [1], when g is of the class C^1, meaning that it is differentiable with continuous first order partial derivatives, then the above conditions are satisfied by applying suitable bounds on the partial derivatives of g. It is possible to show that such bounds can be applied in the case of our epidemic model, from the boundedness of the control and state variables ensuring that Assumptions (a)-(b) are true.

Consider a performance index of the form:

$$\mathcal{F}(u(t)) = \int_0^T f(t,x(t),u(t))dt \tag{0.2}$$

The following theorem, provides the sufficient conditions for the existence of a solution to the optimal control problem.

Theorem 1 *[1] Suppose that assumptions a-b hold, that f is continuous and moreover that:*

1. *The set of solutions to the system equations (0.1) with corresponding control functions in Ω is nonempty.*
2. *Ω is convex.*
3. *f is convex on Ω and g can be written as $g(t,x,u) = a(t,x) + b(t,x)u$.*

Then there exists an optimal control $u^*(t)$ with corresponding optimal trajectory $x^*(t)$ minimizing $\mathcal{F}(u(t))$.

We now prove the conditions of the theorem are satisfied for our model. As noted earlier, conditions (a)-(b) are satisfied. The performance index integrand is

$$f(t,x(t),u(t)) = I_O(t) + I_Y(t) + \frac{W_O}{2}u_O^2(t) + \frac{W_Y}{2}u_Y^2(t) \tag{0.3}$$

which is continuous, and $\Omega = \left\{u(t) \in L^2(O,T)^2 \| a \leq u_O(t), u_Y(t) \leq b, t \in [0,T]\right\}$. We now examine conditions 1-3.

1. From the *Picard-Lindelöf* theorem [2], if the system equations are continuous and Lipschitz and the solutions to the system equations are bounded, then there is a unique solution corresponding to every admissible control in Ω. It is trivial to verify the continuity of the state equations (0.1). Additionally, every element of the variable vector $x(t)$ is bounded by the corresponding age-group totals T_O, T_Y. In other words, $x(t) \in [O, \max\{T_O, T_Y\}]^{16}$ which is a compact and convex set. Finally, the Lipschitz property can be verified with the use of the following lemma from [3].

   Lemma 2 *If the vector function $\mathbf{X}(\mathbf{x}, t)$ is of class C^1 in a compact convex domain D, then it satisfies the Lipschitz condition there.*

2. From the *Heine-Borel* theorem, it follows that Ω is convex because as a closed and bounded set in $\mathbb{R}^2$ [4].

3. The state equations are linearly dependent on the control functions $u_O(t)$ and $u_Y(t)$ so they can be written in the form $g(t, x, u) = a(t, x) + b(t, x)u$. The convexity of the integrand f in the objective functional, follows from the fact that it is quadratic in the control functions.

References

1. Fleming W, Rishel R. Deterministic and Stochastic Optimal Control. Springer-Verlag New York; 1975.

2. Coddington EA, Levinson N. Theory of ordinary differential equations. Tata McGraw-Hill Education; 1955.

3. Birkhoff G, Rota GC. Ordinary differential equations. John Wiley & Sons; 1978.

4. Rudin W, et al. Principles of mathematical analysis. vol. 3. McGraw-hill New York; 1976.
