# Supplementary material for: Optimal age-specific vaccination control for COVID-19: An Irish case study
Source: PLoS One. 2023 Sep 5;18(9):e0290974. doi: 10.1371/journal.pone.0290974 (PMC10479919; doi:10.1371/journal.pone.0290974)
Supplement: S2 Appendix — (PDF) [file pone.0290974.s003.pdf]

Appendix B

Summary of simulation code

The R code associated with this work can be found in on Github: https://github.com/elenizavrakli/Optimal-Age-Specific-Vaccination-Control. We provide two scripts in order to simulate the evolution of the pandemic with and without intervention through vaccination. In both scripts, we use two packages: *deSolve*, which provides methods for solving differential equations, and *tidyverse* which offers an array of tools for data manipulation, visualisation etc.

Baseline situation without vaccination

The script is organised in 6 blocks as follows:

1. **Initialise system states and parameters.** Set initial values for the susceptible, exposed, infectious and recovered population in each age group. Additionally, introduce the refusal rates for the vaccine, the mean holding times for the exposed and infectious states and the R0 numbers which represent the dynamics of transmission amongst age groups. Finally, we set the time frame for the experiment.

2. **Creating state and parameter vectors.** Bring all states together in a named vector using the initial values defined previously, while setting the initial values for all compartments associated with the vaccination equal to 0. Similarly, we define the parameter values used in the model according to the initialisation in the previous step.

3. **Time sequence and control.** Define the time sequence for which to solve the system of ODEs and initialise the control functions to 0. In this case, they will never get updated, since we are exploring the baseline situation where no vaccination is applied.

4. **Define the system of ODEs.** Create a function containing the system of ODEs, as defined in section **??**.

5. **Solve the system of ODEs.** Use the ODE solver provided by the *deSolve* package to obtain the state trajectories. We also obtain the percentage of recovered people for each age group as well as the total percentage of the population that got infected.

6. **Plotting the trajectories.** Plot the trajectories of the susceptible, infected and recovered compartments for both age groups. We also save some of the state vectors to produce comparisons with the case where the optimal vaccination strategy is implemented.

Case where optimal vaccination strategy in place

The script is organised similarly to the previous case. It is organised in 9 blocks as follows:

1. **Initialise system states and parameters.** Same as before, set initial values states, parameters and the time frame for the experiment. There are some additional parameters associated with the vaccination, namely the vaccine effectiveness and mean holding time for the vaccine. Additionally, we define the upper bound for the control functions and the error threshold for the optimisation convergence.

2. **Creating state and parameter vectors.** Same as the previous case. The states associated with the vaccine are again initialised to 0, because we assume the vaccination is only getting started now.

3. **Time sequence and control.** Same as the baseline case. We need two time sequence, one increasing and one decreasing, indicating the solution of the state system forwards in time and the solution of the adjoint system backwards in time respectively. The control functions will get updated after every solution of the state and adjoint systems.

4. **Define the state ODEs.**

5. **Solve the state ODEs forwards in time.**

6. **Define the adjoint ODEs.** Create a function containing the system of adjoint ODEs as obtained through optimal control in section **??**.

7. **Solve the adjoint ODEs backwards in time.**

8. **Repeat process until convergence.** Design a while loop which includes the updates of the control function after each solution of the state and adjoint system. The loop is exited when two consecutive state trajectories are obtained to be closer than the predetermined error threshold. We obtain the percentage of recovered people for each age group as well, the total percentage of the population that got infected, the percentage of vaccinated people for each age group and the total percentage of the population protected from the vaccine.

9. **Plotting the trajectories.** Plot the trajectories of the susceptible, infected and recovered compartments for both age groups. We also plot the control function for each age group and then provide comparison plots for the infectious individuals with and without the optimal vaccination strategy in place, for both age groups.

Our code is designed to simulate the situation in the Republic of Ireland, meaning that we used initial values for the states and parameters, according to estimates of these numbers found in [**?**] and [**?**]. The code is written so that any values can be used, in order to study the effect that they have on the infection dynamics. The versatility of our code allows us to apply this approach to any country by making changes in the two first blocks in both of our R scripts. We can change the values associated with the population, the transmission rates, mean holding times, refusal rate etc.
